# Glutathione S-transferase (GST) and cortisol levels vs. microbiology of the digestive system of sheep during lambing

**DOI:** 10.1186/s12917-022-03201-y

**Published:** 2022-03-18

**Authors:** Natalia Szeligowska, Paulina Cholewińska, Jakub Smoliński, Konrad Wojnarowski, Przemysław Pokorny, Katarzyna Czyż, Krystyna Pogoda-Sewerniak

**Affiliations:** 1 Institute of Animal Breeding, Wrocław University of Enviromental and Life Sciences, Chełmońskiego St. 38C, 51-630 Wrocław, Poland; 2 Present Address: Chair for Fish Diseases and Fisheries Biology, Ludwig-Maximilians-University of Munich, 80539 Munich, Germany; 3 Department of Environmental Hygiene and Animal Welfare, Wrocław University of Enviromental and Life Sciences, Chełmońskiego St. 38E, 51-630 Wrocław, Poland

**Keywords:** microbiome, ruminants, placenta, pregnancy, hypothalamic-pituitary-adrenal axis

## Abstract

**Background:**

During parturition, animals exhibit variation in hormone levels, homeostasis disturbance and dysfunction of the immune system as a result of stress. Glutathione S-transferase (GST) is responsible for the occurrence of oxidative stress in the cells. Cortisol is known as the stress hormone, but it is also involved in the metabolism of proteins, carbohydrates and metabolism processes led by adipose tissue. The aim of the this study was to determine how the levels of GST and cortisol change depending on the parity. Additionally, the influence of lambing on the microbiological composition of the digestive system and placenta in Olkuska sheep was investigated.

**Methods:**

Eighteen ewes were selected for the experiment - primiparas (*n* = 9) and multiparas (*n* = 9), they were kept in the same environmental conditions, had the same diet and did not show any disease symptoms. Fecal samples were collected individually from each ewe (*n* = 18) and then bacterial DNA isolation was made, then qPCR analysis for Firmicutes, Bacteroidetes, Actinobacteria, Proteobacteria phyla and *Lactobacillaceae* family bacteria levels was performed. These samples were also used to analyze cortisol levels by ELISA test. In addition, placenta fragments were collected during delivery, and then the GST level from the tissue was tested.

**Results:**

The analysis of the results showed a higher level of cortisol in primiparous sheep than in multiparous ones, as in the case of glutathione transferase. There were differences between both studied groups in the microbiological composition of the digestive system. In primiparous sheep, the levels of the tested microorganisms were significantly lower than in multiparous ones. A similar relationship occurred in the study of the placental microbiome.

**Conclusion:**

The results show that sheep microbiome, cortisol and GST levels are different in primiparas and multiparas. The study conducted may constitute an introduction to further analyzes that would help positively affect the welfare and homeostasis of the female organism.

## Background

Studies regarding stress factors that adversely affect the maintenance of the homeostasis of the organism are becoming more and more popular among scientists. They are related to, inter alia, the transport of the animal, a sudden change of the living environment, the isolation of individual animals from the herd, as well as adaptation to the physiological state of pregnancy [1, 2]. In mammals, there is two-way communication between the brain and peripheral organs. Stress-inducing stimuli are perceived by the central nervous system (CNS), which affects the functioning of organs and physiological changes within them, while organs can directly affect the CNS. The hypothalamic-pituitary-adrenal (HPA) axis causes the systemic increase in the level of glucocorticosteroids (e.g. cortisol) [1].

The influence of the microbiota-gut-brain axis on the maintenance of the organism’s homeostasis has been studied for several years. The microflora inhabiting the digestive system of ruminants creates a specific ecosystem composed mainly of bacteria, archaea, protozoa and fungi. However, the greatest number is represented by bacteria, mainly from the Firmicutes and Bacteroidetes phyla. Microbes and the brain can communicate with each other in various ways, including through the immune system, the vagus nerve and metabolites such as short-chain fatty acids and peptidoglycans [3, 4].

Apart from diet and age, and individual factors the composition of the digestive system microbiota is also influenced by the physiological state and stress. Changes related to the presence of stressors can also manifest themselves in cells [2, 3, 5]. Among other things, an increase in the level of GST (Glutathione-S-Transferase) indicates the occurrence of cellular stress, and in combination with the level of cortisol, it may indicate the occurrence of a stressful situation for an individual. As a consequence, the composition of the microflora in the digestive system is disturbed [6–9].

Prolonged stress facilitates the colonization and multiplication of pathogenic organisms in the intestines, exposing the body to disease, other effects are hormone disruptions or undesirable behavioral responses during development. Additionally, according to the research by Bailey et al. [10], the effect of stress factors on mice reduced the number of bacteria belonging to the Lactobacillaceae family. It is important to study the effects of stress during pregnancy, as it may adversely affect the development of the fetus and the composition of the microbiome, which plays a key role from the very first days of life, e.g. immune [2, 11, 12].

The aim of the study was to determine how lambing affects the microbiological composition of the digestive system as well as the level of cortisol and GST in primiparous and multiparous ewes of Olkuska sheep.

## Material and methods

### Animals

The Eighteen Olkuska sheep were selected for the study, 9 of which were ewes at the age of 2 years (multiparas) and 9 were 14 months of age (primiparas) – whose selection was based on type of birth (maximum 5 day difference between partiturations and only twin births) and health status. The herd has been kept since 2011 at a research station at the University of Life Sciences in Wrocław, Poland. The animals were in good health conditions and showed no symptoms of disease. They stayed in the same environmental conditions, in an indoor-pasture rearing system, and under the constant care of a veterinarian.

Polish Olkuska sheep is a breed that has a typical structure of dairy animals, large head and ears, and is moderately muscular. The wool is uniformly white, with loose staples. It is also characterized by good maternal instinct, high prolificacy rate, high fertility (200%). Additionally, Polish Olkuska sheep is used for crossbreeding with meat breeds. Since 2005 it has been included in the Genetic Resources Protection Program due to the posessession of genes responsible for high fertility and good adaptation to environmental conditions in Poland [13].

During the breeding season and early pregnancy (up to the 3rd month), the animals received oat grain (300 g / head / day), lupine (a mixture of white and narrow-leaved - 100 g / head / day) and had access to hay ad libitum. Additionally, during the period of preparation for breeding (from August to the end of September), they had access to pasture. The diet that was used in the study herd from the fourth month of pregnancy was based on beet pulp (100 g / head / day), lupine (a mixture of white and narrow-leaved - 600 g / head / day), oat grain (600 g / head / day) and hay ad libitum.

### Sample collection

Fecal samples for testing were collected in three periods: mating, high pregnancy and delivery (up to 24 h after delivery). They were collected from each ewe immediately after defecation (up to 10 s) into sterile 100 ml containers. The placenta fragments were collected immediately after delivery into sterile 100 ml containers. Immediately after collection, all samples were frozen at − 5 °C for transport (10 min), and then stored at − 26 °C for further analysis.

### Bacterial DNA isolation

Genomic Mini AX Stool kit was used to isolate DNA from feses and Genomic Bacteria + kit (A&A Biotechnology, Gdańsk, Poland) for placenta, both of which were modified by addition of mutanolysin (5 μl) and lysozyme (20 μl) for the purposes of performed analyzes. The amount of animal material used for the fecal / tissue analysis was 100 mg which was then placed in 2 ml Eppendorf tubes (tissue was previously homogenized in liquid nitrogen). Then the isolation was performed according to the protocols included in kits.

After isolation, the quality of the obtained material was assessed using a NanoDrop 2000 spectrophotometer (Thermo Scientific, Waltham, MA, USA). The DNA content for the stool was 100 μg / μl and for the placenta: 450 μg / μl. In both cases, the contamination level of the samples was 2.0–2.2 for parameter 260/230 and 1.8–2.0 for parameter 260/280.

### qPCR analysis

qPCR analysis was performed using a BIO - RAD CFX Connect 96 Touch instrument (Bio-Rad Laboratories, Inc., California, USA). For this purpose, the SsoAdvanced™ Universal SYBR® Green Supermix kit (Bio-Rad Laboratories, Inc., California, USA) was used. Three technical replicates were prepared in a volume of 10 μl (Tab. 1). For each tested amplicon, a No Template Control (NTC) test was performed, in which no DNA was added (analysis of primer amplification) - to check the amplification performance of the primers.

**Table 1:** Mix Ratio to qPCR [14]

Component	Volume in a 10 μl reaction
Primer (F + R)	1 μl (0.8 μM)
DNA matrix	2 μl (0.04–0.015 × 10ˉ^4^)
SsoAdvanced™ Universal SYBR® Green Supermix	5 μl
Sterile water	2 μl

To carry out amplification by means of qPCR, reference genes characteristic for the studied groups of bacteria were used, which were compared with the amplicon specific for all bacteria (Tab. 2).

**Table 2:** qPCR Primers [15–19]

NAME	FORWARD (5′-3′)	REVERSE (5′-3′)
UNIVERSAL EUBACTERIAL GENES [14]	530F (5′-GTC CCA GCM GCN GCG G-3′)	100R (5′-GGG TTN CGN TCG TTG-3′)
FIRMICUTES [15]	928F-Firm (5′-TGA AAC TYA AAG GAA TTG ACG-3′)	1040FirmR (5′-ACC ATG CAC CAC CTG TC-3′)
BACTEROIDETES [15]	98cfbF (5′-CRA ACA GGA TTA GAT ACC CT-3′)	cfb967R (5′-GGT AAG GGT TCC TCG CGT AT-3′)
LACTOBACILLACEAE [16]	Lac1 forward (5′-AGC AGT AGG GAA TCT TCC A-3′)	Lac2Seq (5′-ATT TCA CCG CTA CAC ATG-3′)
PROTEOBACTERIA [17]	27F (5′-GAGTTTGATCMTGGCTCAG-3′)	529R (5′ CAKAAAGGAGGTGATCC-3′)
ACTINOBACTERIA [18]	Act1159R (5′-TCCGAGTTRACCCCGGC-3′)	b338F (5′-ACGGGCGGTGTGTACA-3′)

For the development of a standard curve, a series of 10^− 2^ to 10^− 7^ dilutions were made to determine the performance of the amplicons tested. A 10^− 6^ dilution was selected for further analyzes. qPCR was performed according to the protocol consisting of 40 cycles: polymerase activation and DNA denaturation 95 °C (3 min), denaturation 95 °C (15 s), annealing 60.5 °C (15 s), extension and plate reading at 72 °C (40 s). The analysis of the melting curves for the samples was carried out at temperatures ranging from 65 °C (5 s) to 95 °C (increments of 0.5 °C every 2 s).

The data obtained was processed using CFX Maestro software (Bio-Rad Laboratories, Inc., Irvine, CA, USA). The performance of individual primers was correct and in line with the standards established by BIO-RAD. It was 89.4% for the Firmicutes phylum, 100.9% for Bacteroidetes, 91.6% for Actinobacteria, 94.1% for Proteobacteria, 95.4% for Lactobacillaceae 98.4% and for the Universal Eubacterial Gene 94.4%. A sample with a DNA level of 100 μg / μl and impurities at the level compliant with the above-mentioned standards was an arbitrary calibrator [20].

### GST analysis

The samples were homogenized with liquid nitrogen and unified with respect to mass to 615.6 μg (sd = 2.1). Then, using Glutathione-S-transferase (GST-ST) Activity Assay Kit (ElabScience®, China) and CARY 100 Conc UV-Vis spectrophotometer (Varian, Australia), the level of GST - ST was evaluated according to the protocol. Absorbance was measured at 340 nm. The analysis was performed in 3 replications. The results obtained in accordance with the protocol were determined in U/ mg protein in tissue.

### Cortisol analysis

Analysis of the level of cortisol in the fecal supernatant (previously suspended in PBS at a ratio of 1:9) was performed with the Sheep Cortisol Elisa kit (Bioassay Technology Laboratory, Shanghai, China) according to the manufacturer’s protocol. Each measurement was repeated 3 times.

### Statistical analysis of the results

The obtained results were analyzed with Statistica ver. 13.1. The data distribution was checked with the Shapiro-Wilk test. Analysis of the results was performed using of ANOVA (with repeat measurments; factor: numbers of parturitions). Addictionally was made Spearman correlation between cortisol and GST level. The differences were determined using the Tukey test (*P* ≤ 0.05).

## Results

**Fig. 1:**
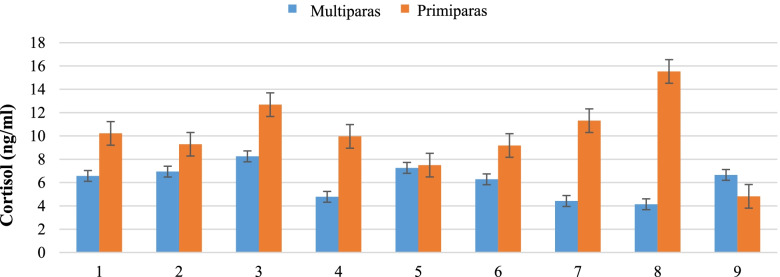
Individual fecal cortisol level in primiparous and multiparous ewes

The analysis of the level of cortisol in the fecals on the day of delivery showed a significant differentiation depending on the parity. In the case of the primiparas, this level was significantly higher than in multiparous ewes (16.96 vs. 6.14 ng/ml, respectively; *p* = 0.0019). Additionally, when analyzing the differences taking into account the individual results (Fig. 1), there is a visible trend of higher cortisol levels in the primiparous ewes.

**Fig. 2:**
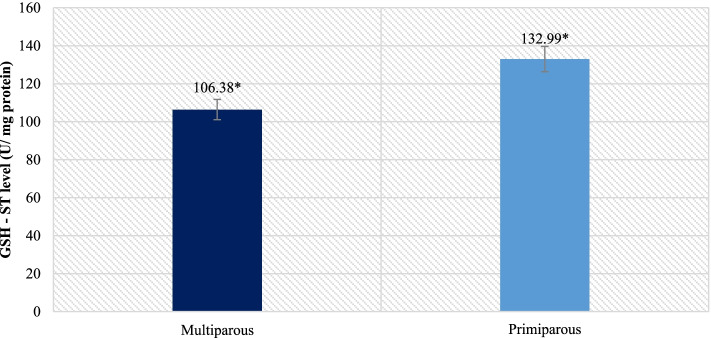
Level of GST – ST in placenta (* *P* ≤ 0.05)

However, in the analysis of GST-ST levels, differences were shown as in the case of cortisol levels (Fig. 2). Primiparous ewes were characterized by a significantly higher level of GSH - ST in the placenta compared to multiparous ones (*p* = 0.0459). Additionally, a statistically significant positive correlation (*r* = 0.54, *p* = 0.0345) was demonstrated between the level of cortisol in the fecals and GST - ST in placenta.

**Fig. 3:**
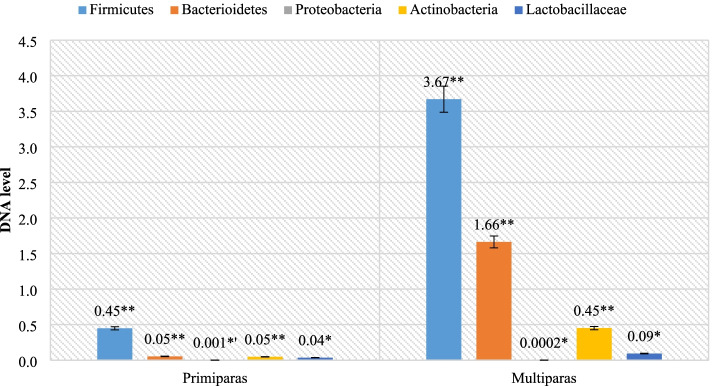
Level of selected bacteria in fecal samples (** *P* ≤ 0.01;* *P* ≤ 0.05)

Then, the level of bacterial DNA of selected bacteria in the stool was analyzed. The obtained results showed significant differences in composition between primiparous and multiparous females. Multiparous ewes were characterized by a much higher level of the studied groups of bacteria compared to the primiparous ones. The exceptions were microorganisms belonging to the Proteobacteria phylum (0.001 and 0.0002, respectively; *p* = 0.0008), which is also shown in Fig. 3.

**Fig. 4:**
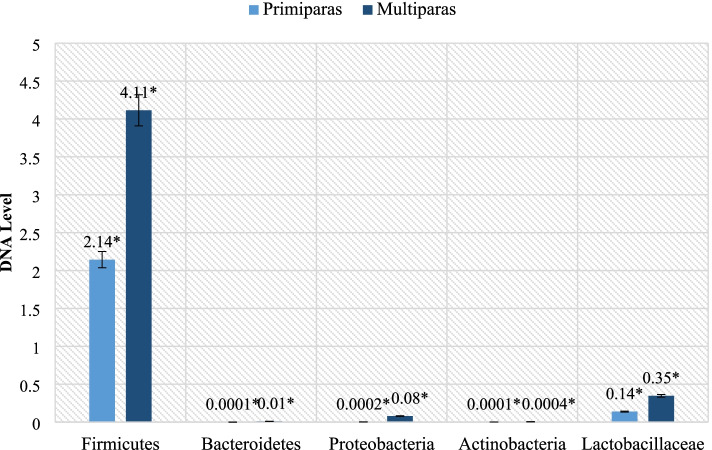
Differences in levels of tested bacteria in placentas of primiparas and multiparas (** P ≤ 0.01;* P ≤ 0.05)

Additionally, the levels of the tested bacteria in the placenta were analyzed. As in the case of fecals, a significant variation in the levels of the bacteria tested was found between primaparas and multiparas (P ≤ 0.05). Overall, a higher level of the studied groups was demonstrated in multiparous ewes compared to the primiparous ones, as shown in Fig. 4.

## Discussion

Microbiological composition of the digestive system of sheep is influenced by factors such as diet, pregnancy, individual factors, stress and age. Ruminants, due to the multi-chamber structure of the stomach (rumen, reticulum, omasum, abomasum), are characterized by the presence of a large population of microorganisms that facilitate the digestion of food of plant origin [1, 3, 20].. The microbiological analyzes of fecal performed in this study showed that the multiparous ewes are characterized by a higher level of the tested bacteria in comparison to the primiparous ones. The exception was bacteria belonging to Proteobacteria, where the content was similar in both cases.

Significant differences in the studied groups of sheep suggest that the composition of the microbiome is influenced by sex hormones, i.e., estrogens (estrone, estriol, estradiol) [21–24]. They contribute to the maturation of the genital organs, lipid metabolism, absorption of nutrients from the gastrointestinal tract, as well as the variability in the population of bacteria colonizing intestines [21–25]. The study by Fuhrman et al. [26] on human confirm that the composition and diversity of the intestinal microflora is related to estrogen metabolism. Especially with the Ruminococcaceae family, belonging to the Clostridia class, while the Bacteroidetes phylum was negatively associated with the metabolism of sex hormones. In the study conducted, the level of these microorganisms in the placenta was very low. On the other hand, their value in fecal was much higher in multiparous than in the primiparous ewes.

For some time it was considered that the uterus of pregnant animals was a sterile environment free from any microorganisms [27, 28]. Bacterial colonization of the endometrium is a natural phenomenon. As a model organism, sheep are used to research the physiology of the placenta, the course of pregnancy and its effect on the mother’s body [27]. The obtained results are successfully transferred to humans. The human uterus has a small, non-pathogenic microbiome composed of representatives of the following phyla: Firmicutes, Proteobacteria, Bacteroidetes, Fusobacteria and Tenericutes [26]. Placental colonization occurs through the transfer of microorganisms through the bloodstream. Study on mice by Jimènez et al. [29] demonstrated that oral bacteria enter the uterine environment through the bloodstream and can affect the parturition. In turn, according to Owens et al. [30] bacteria also have the ability to penetrate the lymphatic system. In addition, the colonization of the placenta in cows takes place through migration from the lower genital tract or from the mother’s intestines during periods of intestinal hyperpermeability [30]. In microbiological analyzes of cows placenta, bacteria belonging to the Proteobacteria, Actinobacteria, Bacteroidetes and Firmicutes phyla were the most abundant. On the other hand, a lower level was observed for the genus Tenericutes and the phyla of Cyanobacteria and Verrucomicrobia. In the study conducted, we demonstrated that in the placenta of sheep there are traces of bacteria belonging to Proteobacteria, Bacteroidetes and also Actinobacteria. The most numerous are microorganisms belonging to the Firmicutes phylum. These results confirm the thesis that the placenta in ruminants is not a sterile environment.

The prenatal period is the time when the organs and systems of the young body are formed. It is important to maintain maternal homeostasis so that the fetus can develop properly. One of the factors that disturb homeostasis is stress [1, 31]. It increases the susceptibility to infections by modulating the composition of the microbiome in the digestive system. In sheep, as in humans, the adrenal gland secretes more cortisol during pregnancy. This hormone may have toxic effects on the fetus and mother [1, 31, 32]. Additionally, the level of cortisol increases with the course of pregnancy, as well as during changes in the metabolism of the ACTH protein, produced by the pituitary gland. This leads to an increase in the concentration of corticotropin (CRH) in the placenta and also affects the time of delivery. The level of cortisol in the sheep’s body is also influenced, among others, by lactation, veterinary procedures (abortions), improper nutrition, miscarriages and the occurrence of diseases (pregnancy toxemia) [33–36]. In our study, we found a higher level of cortisol in primiparous than in multiparous ewes. The data obtained indicate that the sheep in subsequent parity have lower levels of stress hormones, which may be related to the experience acquired during previous lambings. For primiparas it is an unknown experience, and also uncontrolled phenomenon. They do not have a developed pattern of conduct and awareness of the duration of the puerperium. In addition, parturition means, both for animals and humans, extreme physical and mental stress, which explains the increase in cortisol levels [37].

Glutathione transferase (GST) is a protein responsible for reducing oxidative stress in tissues [38]. Its functions also include the synthesis of steroids, conjugation and detoxification of endogenous and exogenous electrolytes in cells. The presence of a higher concentration of this protein in the placenta is related to the protective function of this tissue during pregnancy [38, 39]. During the research, primiparous sheep showed a higher concentration of GST compared to multiparous ones. This result indicates a lower level of oxidative stress in the cells of the tested animals. Glutathione transferase interacts with the metabolism of reactive oxygen species (ROS). The increase in ROS is responsible for the occurrence of oxidative stress in the cells, which leads to a decrease in the level of GST [40, 41]. The consequence of this is the appearance of ketone bodies in the animal and the possibility of inducing pre-eclampsia [40, 41].

## Conclusion

The study conducted shows differences in the level of cortisol and GST in sheep depending on the parity. In primiparous ewes, parturition results in the occurrence of greater stress (measured by the level of cortisol) and the level of glutathione transferase, which may indicate cellular stress. There were also differences in the content of the tested microorganisms in the fecals of both research groups, as well as in the placenta. These results prove that the placenta is not sterile during pregnancy and that microorganisms can be passed on to the offspring. This study should be extended to explore other bacterial phyla and families inhabiting the digestive system of ruminants and the placenta to better understand this complex ecosystem. In addition, further analyzes in this direction are indicated in order to identify trends related to GST and cortisol levels.

### Acknowlegments

The authors would like to thank Magdalena Wołoszyńska (Department of Genetics, Wroclaw University of Environmental and Life Sciences, 51–630 Wroclaw, Poland) for the access to equipment and valuable advice.

### Authors’contributions

Conceptualization, N.S. and P.C.; methodology, N.S., P.C., J.S., K.P.,; validation, K. W, J.S.;formal analysis, N. S, P.C.; investigation, N. S, P.C., K.W.; writing—original draftpreparation, N.S.; writing—review and editing, K.W., K.C; supervision, K.C., P.P. All authors have read and agreed to the published version of the manuscript.

## Data Availability

All data generated or analysed during this study are included in this published article.

## Abbreviations

GST: Glutathione S-transferase
DNA: Deoxyribonucleic acid
qPCR: Polymerase Chain Reaction
NTC: No Template Control
INRA: Institut National de la Recherche Agronomique
RNE: Relative Normalized Expression
